# Optical Absorption and Raman Scattering in ZnO/Mg_*x*_Zn_1−*x*_O Quantum Wells Under Non-Resonant Laser Effect

**DOI:** 10.3390/nano16040276

**Published:** 2026-02-21

**Authors:** S. Uran-Parra, J. A. Gil-Corrales, J. A. Vinasco, A. L. Morales, C. A. Duque

**Affiliations:** 1 Grupo de Materia Condensada-UdeA, Instituto de Física, Facultad de Ciencias Exactas y Naturales, Universidad de Antioquia UdeA, Cl 70 No. 52-21, Medellín 050010, Colombia; salomon.uran@udea.edu.co (S.U.-P.); alvaro.morales@udea.edu.co (A.L.M.); 2 Facultad de Ciencias, Instituto Tecnológico Metropolitano (ITM)-Institución Universitaria, Campus Fraternidad, Calle 73 No. 76A-354 Vía al Volador, Medellín 050034, Colombia; johngil1275@correo.itm.edu.co; 3 Departamento de Ciencias Básicas, Facultad de Ingeniería y Administración, Universidad Nacional de Colombia Sede Palmira, Palmira 763533, Colombia; javinascos@unal.edu.co

**Keywords:** ZnO/MgZnO quantum wells, non-resonant intense laser, linear optical absorption, Raman differential cross section, Raman gain

## Abstract

The influence of a non-resonant intense laser field on the optical absorption and Raman scattering processes in ZnO/Mg_0.2_Zn_0.8_O quantum wells is theoretically investigated. It is shown that the dressing field significantly modifies the confinement potential and reshapes the electronic wave functions, leading to tunable shifts in intersubband transition energies and changes in the dipole matrix elements. These laser-induced effects produce notable variations in the absorption spectrum and strongly modulate the Raman differential cross section and Raman gain. Under the application of a non-resonant laser field, the Raman gain is enhanced by almost a factor of four, whereas off-resonant pumping results in much weaker, yet still field-dependent, responses. The results demonstrate that intense laser fields provide an effective tool to dynamically control the optical and Raman properties of ZnO-based quantum well structures.

## 1. Introduction

Semiconductor quantum wells (QWs) are of high importance in the investigation of nanophotonics and optoelectronic devices due to their quantized electron states and highly tunable optical properties. Among the multiple QW systems that can be found in the literature, ZnO-based heterostructures have received great attention in recent decades due to their prominent optical properties in the blue–ultraviolet (UV) region of the spectrum [1,2,3], wide bandgap semiconductivity, advantages in fabrication, native defects [4], large exciton binding energy [5], and other thermal, optical, and electronic properties.

ZnO-based heterostructures are particularly attractive due to their wide bandgap, strong polarization fields, and excellent suitability for intersubband optical processes. These characteristics make such systems promising for a wide range of applications in electronics [6,7,8] and optoelectronics [9,10]. The bandgap of these heterostructures [11] can be tuned from 3.37 eV in ZnO up to 7.8 eV in MgO. Moreover, Mg incorporation induces minimal lattice distortion while enabling effective control of the carrier concentration and a significant reduction in leakage current [12].

Among ZnO-based heterostructures, ZnO/Mg_*x*_Zn_1−*x*_O QWs are of typical interest due to their confinement properties, which allow an increase in the concentration of localized 2D electron gas [13] and enhanced electron mobility (up to 20 cm^2^/Vs) [14]. These structures are effectively produced through multiple growth methods, such as molecular beam epitaxy, thermal vapor deposition, and pulsed laser deposition [15,16,17], and they present a very important feature: ZnO/MgZn_*x*_O_1−*x*_ QWs exhibit significant spontaneous and piezoelectric polarization along the c-axis [18,19,20], which strongly modifies the confinement potential due to these built-in electric fields.

Previous studies have analyzed the electronic properties of confined semiconductor nanostructures using the effective mass Schrödinger equation under different external conditions [21,22]. However, those works did not address optical scattering processes, particularly Raman emission in ZnO-based QWs under intense laser fields, which remains unexplored.

As a result, there has been a comprehensive study of the effects of external laser fields across multiple heterostructure systems. Specifically, the study of non-resonant intense laser fields (ILFs) has become a major focus of recent research in condensed matter, mainly due to the tailoring of optical and electronic properties resulting from the tunability of the confinement potential along the laser polarization direction [23]. For example, Wang et al. in 2021 [24] reported the tailoring effect of external fields via a terahertz ILF on the shallow-donor-related photoionization cross section in GaAs bulk semiconductors. Donado et al. in 2025 [25] reported the particular effect of combining an external electric field and an ILF on the electronic properties of a truncated cone quantum dot, in which the quantum confinement can be controlled by the dressing parameter $\alpha_{0}$. Additionally, Dagua et al. in 2025 [26] reported the effects of ILF on the probability of electronic transmission in QW AlGaAs/GaAs resonant tunneling diodes based on the Pösch–Teller potential.

All effects on the quantum-confinement properties of a heterostructure will have a noticeable impact on optical absorption, which plays a key role in characterizing these systems. In the case of the ZnO-based heterostructures, Tang et al. in 2025 [27] investigated the generation of double peaks in the interband and intersubband optical absorption in Mg_*x*_Zn_1−*x*_O/Al_*y*_Ga_1−*y*_N core–shell nanowires, depending on the core radius and different crystal phases, while Gu et al. in 2022 [28] reported the shift of the peak positions in the total optical absorption coefficient in MgZnO/ZnO double QWs due to the variation of external electric and magnetic fields. As an additional literature source, Andrzejewski et al. in 2021 [29] conducted experimental measurements and theoretical comparisons of the photoluminescence spectra of multiple asymmetric QWs grown with different widths and internal electric fields, accounting for single-particle transitions and temperature-dependent localized excitonic contributions to photoluminescence. Lastly, Atic et al. in 2024 [30] showed numerically how the Mg concentration and doping density affect the intersubband absorption peak and tunneling current in different configurations of multiple ZnO/MgZnO QWs and resonant-tunneling structures in order to mimic the active regions of a quantum cascade laser.

Quantum confinement modifies light—matter interaction and gives rise to inelastic photon—electron scattering processes (Raman scattering) [31,32]. The electron Raman response is commonly described in terms of the differential cross section (DCS), which provides information about the subband structure and carrier distribution in heterostructures. Previous works have analyzed the influence of structural parameters and external fields on the DCS in semiconductor nanostructures, including ZnO/MgZnO quantum wells and related systems [33,34].

An important quantity characterizing the Raman process is the gain coefficient, which describes the amplification of an optical probe through stimulated Raman scattering under strong optical pumping. Since the Raman gain is proportional to the imaginary part of the third-order susceptibility, it is highly sensitive to nonlinear effects and confinement conditions. Previous studies have shown that structural asymmetry, band non-parabolicity, and external perturbations can significantly modify the Raman gain in semiconductor nanostructures [35,36].

Raman scattering in ZnO-based nanostructures has been experimentally and theoretically investigated, revealing that the spectral features depend strongly on the system’s confinement and electronic structure [37,38]. These studies demonstrate the relevance of quantum confinement to the Raman response; however, they consider static structures and do not include external laser-dressing effects that can dynamically modify the effective potential profile. Consequently, the influence of a non-resonant intense laser field on Raman emission in ZnO/MgZnO QWs remains an open problem.

Non-resonant intense laser fields are known to modify the effective confinement potential through the Kramers–Henneberger transformation, producing controllable changes in the electronic structure and optical transition matrix elements. However, their impact on Raman scattering efficiency and stimulated Raman gain in polar wide-bandgap quantum wells has not yet been systematically analyzed.

In this work, we theoretically investigate linear optical absorption, Raman differential cross section, and Raman gain in a ZnO/Mg_0.2_Zn_0.8_O QW including internal polarization fields under a non-resonant intense laser field. By combining the envelope-function approximation with a laser-dressed potential, we demonstrate that the dressing parameter enables strong control over transition energies, wave-function symmetry, and nonlinear optical response. The results reveal a substantial enhancement and tunability of Raman gain, providing a mechanism for external optical control of Raman amplification in oxide QW structures.

The paper is organized as follows: In Section 2, we schematize the system and the theory, including the main equations used for the dressed potential, optical absorption, DCS, and Raman gain. In Section 3, we present and discuss our main results. Finally, in Section 4, we present our concluding remarks.

## 2. Theory

### 2.1. ZnO/Mg_x_${\mathrm{Zn}}_{1-x}$O Heterostructure

**Figure 1:**
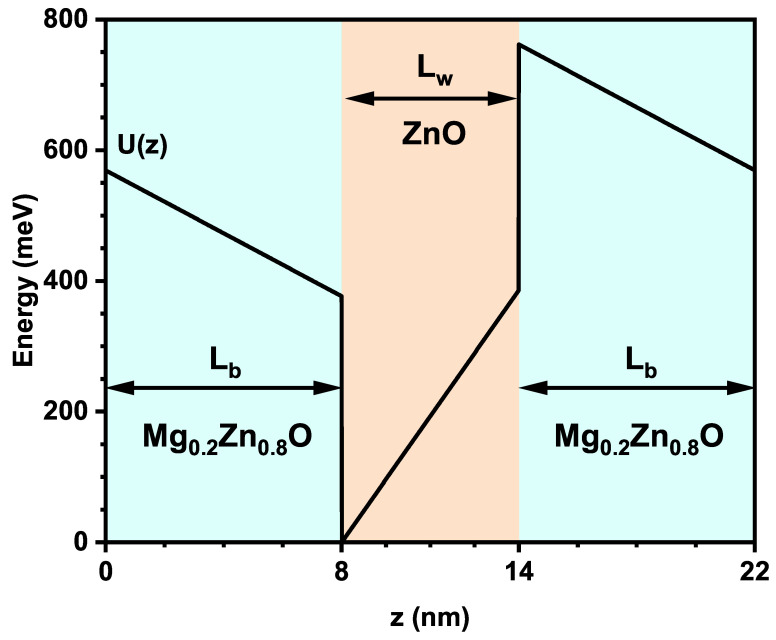
Potential profile U(z) represented in the ZnO/Mg_0.2_Zn_0.8_O QW system with dimensions $L_{w}=6$ nm and $L_{b}=8$ nm.

The system consists of an asymmetric quantum well, where the well (ZnO) and the barriers (Mg_*x*_Zn_1−*x*_O) have dimensions of $L_{w}=6$ nm and $L_{b}=8$ nm, respectively, and the concentration of Mg has been fixed at x=0.2; see Figure 1. Following [39], the total potential *U* consists of two parts, the band offset and the built-in electric fields resulting from piezoelectric and spontaneous polarization, and is given by the following:

(1) $$U\left(z\right)=\left\{\begin{array}{cc} eF_{b}z+\Delta E_{c}\;, & 0<z<L_{b}\;,\\ eF_{w}z+e\left(F_{b}-F_{w}\right)L_{b}\;, & L_{b}<z<L_{b}+L_{w}\;,\\ eF_{b}z+e\left(F_{w}-F_{b}\right)L_{w}+\Delta E_{c}\;, & L_{b}+L_{w}<z<L\;, \end{array}\right.$$

where *z* is the direction of growth of the structure, $F_{w}$ and $F_{b}$ are the values of the built-in electric fields within the well and the barrier, respectively, $\Delta E_{c}$ is the discontinuity of the conduction band, and *L* is the total length of the system. These internal electric fields are calculated using the following equation:

(2) $$F_{w}=\frac{2L_{b}P_{\mathrm{tot}}}{2\varepsilon_{b}L_{b}+\varepsilon_{w}L_{w}}\;,\;F_{b}=-F_{w}\frac{L_{w}}{2L_{b}}\;,$$

where $P_{\mathrm{tot}}$ is the total contribution from piezoelectric and spontaneous polarization in the entire system, and $\varepsilon_{w,b}$ are the dielectric constants for the well and barrier, respectively. The band offset is introduced as a fraction of the bandgap difference across the heterojunction. Here, the conduction band discontinuity is defined as follows:

(3) $$\Delta E_{c}=0.75\;\left(E_{g,{\mathrm{Mg}}_{x}{\mathrm{Zn}}_{1-x}O}-E_{g,\mathrm{ZnO}}\right)\;,$$

corresponding to a 75% conduction band offset, consistent with reported values for ZnO/Mg_*x*_Zn_1−*x*_O systems [39,40,41]. The band offset ratio $Q=\Delta E_{c}/\Delta E_{g}=0.75$ was not arbitrarily adopted but corresponds to the widely reported conduction-band dominance in ZnO/MgZnO heterostructures. Experimental and theoretical studies consistently indicate that the band discontinuity in MgZnO alloys occurs mainly in the conduction band, typically between 70% and 80% of the total band gap difference, depending on Mg composition and growth conditions. Therefore, the selected value lies within the experimentally supported interval and serves as a standard approximation for modeling intersubband transitions in ZnO-based quantum wells.

Small variations of this parameter mainly produce rigid shifts in subband energies without altering the qualitative behavior of the Raman gain, which will be discussed in this work.

The multiple parameters used for the heterostructure, including dependencies on Mg concentration, internal electric fields, and band discontinuity values, are listed in Table 1. The corresponding values of ZnO can be obtained by putting x=0.

### 2.2. Non-Resonant Intense Laser Field (ILF) and Schrödinger Equation

In the presence of a non-resonant intense laser field (ILF), the interaction between the confined electron in the heterostructure and the incident electromagnetic field can be treated semi-classically, making use of the Kramers–Henneberger transformation, which transfers the time-dependence of the Hamiltonian to the confinement potential through an oscillating argument [44]. When a high-frequency incident field is considered relative to the proper frequencies of the system’s transitions, the time-dependent potential can be approximated as a laser-dressed potential, obtained by averaging over a period of the oscillating potential. In this context, the Schrödinger equation for the electron in the QW, under the envelope function approximation, is given by the following [45]:

(4) $$-\frac{{ћ}^{2}}{2}\frac{\partial }{\partial z}\left(\frac{1}{m^{*}\left(z\right)}\frac{\partial \psi_{j}\left(z,\alpha_{0}\right)}{\partial z}\right)+V\left(z,\alpha_{0}\right)\;\psi_{j}\left(z,\alpha_{0}\right)=E_{j}\left(\alpha_{0}\right)\;\psi_{j}\left(z,\alpha_{0}\right)\;,$$

where *ћ* is the reduced Planck constant, $m^{*}\left(z\right)$ represents the effective mass varying along the *z* direction, and $\psi_{j},E_{j}$ are the wave function and energy associated with the *j*th state for the electron in the system. Under the approximation mentioned, the laser-dressed confinement potential *V* obtained from the interaction of the system with the external laser field is given by the following:

(5) $$V\left(z,\alpha_{0}\right)=\frac{1}{T}\int_{0}^{T}U\left[z+\alpha_{0}\;\sin\left(\omega_{0}t\right)\right]dt\;,$$

where $\alpha_{0}$ is the dressing laser parameter and $\omega_{0}$ is the non-resonant frequency of the laser field.

The effective mass approximation with parabolic dispersion is valid as long as the electronic energies involved remain close to the conduction band minimum. In ZnO QWs, band non-parabolicity becomes relevant only for carrier energies of several hundreds of meV above the band edge.

In the present work, the relevant electronic scale is the intersubband transition energy, which is more than one order of magnitude smaller than the energy range where non-parabolicity becomes significant. Moreover, within the Kramers–Henneberger transformation, the intense laser field modifies the confinement potential via spatial dressing, while the kinetic-energy operator remains unchanged. Therefore, the field renormalizes the eigenstates but does not accelerate electrons to high kinetic energies where non-parabolic corrections would be required. Consequently, the parabolic effective mass approximation provides an accurate description of the electronic structure for the considered regime.

### 2.3. Linear Optical Absorption Coefficient and Temperature Dependence

**Figure 2:**
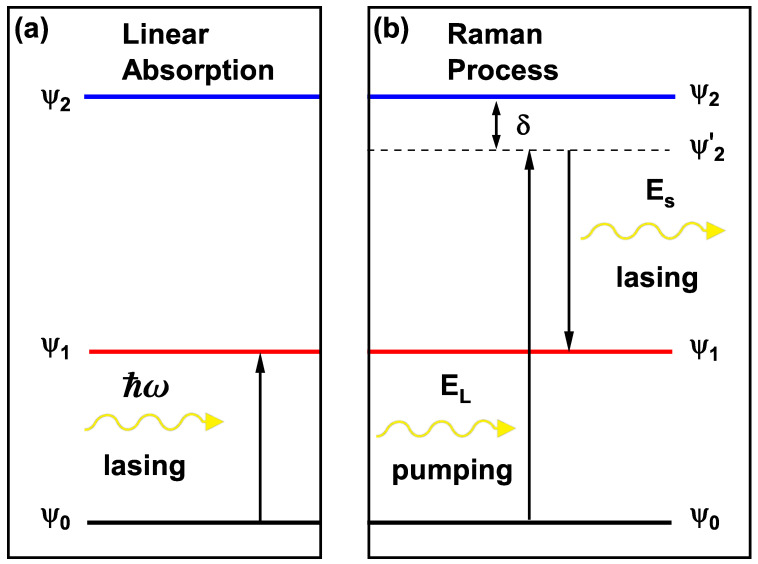
Transitions in the linear optical absorption and Raman process. In (**a**), the states $\psi_{0}$ and $\psi_{1}$ for a given value of the incident photon’s energy ћω are represented. In (**b**), the following are depicted: the Stokes Raman process of optical pumping from $\psi_{0}$ to a virtual state $\psi_{2}^{\prime }$ due to the incident radiation $E_{L}$, and the lasing of secondary radiation $E_{S}$ to reach $\psi_{1}$, in addition to the pump detuning δ between the real $\psi_{2}$ and virtual $\psi_{2}^{\prime }$ states.

The absorption of an incident photon’s energy can produce a transition between $\psi_{i}$ and $\psi_{j}$ states (Figure 2a). This process in the system is modeled as a semi-classical interaction, where the quantum discrete states are the electronic states and the incident electromagnetic field behaves classically, allowing each electron to interact with the field and be excited to higher energy levels. Employing a matrix density formalism combined with perturbation theory, it is possible to obtain an expression for the first-order susceptibility [44], and from this, the standard formula for linear optical absorption, which is given by the following [46]:

(6) $$\alpha_{ij}^{\left(1\right)}\left(\omega \right)=ћ\omega \frac{\Lambda_{ij}}{\tau_{\mathrm{in}}}\frac{|M_{ij}{|}^{2}}{{\left(E_{j}-E_{i}-ћ\omega \right)}^{2}+{\left(ћ/\tau_{\mathrm{in}}\right)}^{2}}\;,$$

where $\Lambda_{ij}=\sqrt{\frac{\mu_{0}}{\varepsilon_{w}\varepsilon_{0}}}e^{2}\sigma_{ij}$, ω is the frequency of the incident photon, $M_{ij}=\langle \psi_{i}\left|z\right|\psi_{j}\rangle$ is the transition matrix element, $\sigma_{ij}$ is the difference of volumetric carrier density between the states $\psi_{i}$ and $\psi_{j}$, respectively, $\tau_{\mathrm{in}}$ is the relaxation time between bands, and $\varepsilon_{0},\mu_{0}$ are the vacuum permittivity and permeability, respectively.

Since optical absorption depends on the volumetric carrier densities, it is important to understand and account for effects from the laser-dressed potential and intensive properties, such as temperature, on the populations of the ground and excited states. The total superficial carrier density $n_{d}$ in the system is related to temperature through the superficial carrier density for the $\psi_{i}$ state $n_{i}$ as $n_{d}=\sum_{i=0}^{2}n_{i}$, where $n_{i}$ is given by the following [46]:

(7) $$n_{i}=\frac{m_{w}^{*}}{\pi {ћ}^{2}}\int_{E_{i}}^{\infty }\frac{dE}{1+\exp\left(\frac{E-E_{f}}{k_{B}T}\right)}\;,$$

where $E_{i}$ is the energy of the state $\psi_{i}$, $k_{B}$ is the Boltzmann constant, $m_{w}^{*}$ is the effective mass of the well, and the relation between $n_{d}$ and $n_{i}$ is used to obtain the Fermi level $E_{f}$ at temperature *T*. With the Fermi level known, the difference in the volumetric electronic population between states *i* and *j* $\sigma_{ij}$ can be calculated as follows:

(8) $$\sigma_{ij}=\frac{m^{*}k_{B}T}{\pi {ћ}^{2}L}\ln\left(\frac{1+\exp[\left(E_{f}-E_{i}\right)/\left(k_{B}T\right)]}{1+\exp[\left(E_{f}-E_{j}\right)/\left(k_{B}T\right)]}\right)\;.$$

### 2.4. Raman Gain Theory

For scattering in general, an important concept is the differential cross section (DCS), which for the case of Raman scattering is given by the following [45]:

(9) $$\frac{d^{2}\sigma }{d\Omega \;d\nu_{S}}=\Theta \left(\nu_{L},\nu_{S}\right)\;W\left(\nu_{S},{\hat{u}}_{S}\right)\;,$$

where σ is the cross section, Ω is the solid angle, $\Theta \left(\nu_{L},\nu_{S}\right)=\frac{V^{2}\;\nu_{S}\;n\left(\nu_{S}\right)}{8\pi^{3}\;c^{4}\;n\left(\nu_{L}\right)}$, *c* is the vacuum speed of light, $n(\nu_{L,S})$ are the frequency-dependent refractive indices, which can be considered equal, $\nu_{L}$ is the frequency of incident radiation, $\nu_{S}$ is the frequency of secondary radiation emitted, ${\hat{u}}_{S}$ is the polarization vector for the secondary radiation field emitted, and $V=\frac{2}{\sigma_{01}}$ is the volume of the QW.

The Raman process considers two transitions: $\psi_{0}\to \psi_{2}$ of absorption and $\psi_{2}\to \psi_{1}$ of emission (Figure 2b), for which the transition rate $W(\nu_{S},{\hat{u}}_{S})$ is as follows [45]:

(10) $$W\left(\nu_{S},{\hat{u}}_{S}\right)=\frac{2}{ћ}\frac{T_{021}^{2}}{{\left(E_{s}+E_{1}-E_{2}\right)}^{2}+\Gamma_{a}^{2}}\times \frac{\Gamma_{f}}{{\left(E_{L}-E_{s}+E_{0}-E_{1}\right)}^{2}+\Gamma_{f}^{2}}\;,$$

where $T_{021}=\langle \psi_{0}\left|H_{L}\right|\psi_{2}\rangle \times \langle \psi_{1}\left|H_{S}\right|\psi_{2}\rangle$ and $\Gamma_{a},\Gamma_{f}$ are the broadenings of the intermediate and final states, which are chosen to be $\Gamma_{a}=\Gamma_{f}=5\;\mathrm{meV}$ [33]. The parameter $\Gamma_{a}$ ($\Gamma_{f}$) represents the homogeneous broadening associated with the finite electronic dephasing time (Γ=ћ/τ), determined by electron–phonon interaction, interface roughness, and impurity scattering in polar QWs. Experimental studies of ZnO/MgZnO QWs report emission linewidths of several tens of meV, due to a combination of homogeneous and inhomogeneous mechanisms [47], dominated by LO-phonon coupling and defect scattering. The homogeneous contribution relevant for Raman processes is typically one order of magnitude smaller, leading to characteristic dephasing broadenings in the meV range. Therefore, the chosen value $\Gamma_{a}=5$ meV lies within the physically expected interval for polar ZnO quantum wells. It should be noted that the variation in the value of the $\Gamma_{a}$ ($\Gamma_{f}$) parameter does not affect the position of the DCS peaks and their displacement remains unchanged; the changes are only reflected in the amplitude of the peaks.

Since the problem is in one dimension, the polarization vector for both incident and emitted secondary radiation can be taken as ${\hat{u}}_{L}={\hat{u}}_{S}=\left(1,0,0\right)$ on the *z*-axis, and then the photon–electron interaction operators $H_{k}$ are given by the following:

(11) $$H_{k}=-\frac{i|e|}{m_{0}}\sqrt{\frac{2\pi {ћ}^{3}}{V\nu_{k}}}\frac{\partial }{\partial z}\;.$$

The magnitude of the differential cross section can be explained in terms of the expected value of the velocity operator $P_{ij}=\langle \psi_{i}\left|\frac{\partial }{\partial z}\right|\psi_{j}\rangle$ between the $\psi_{i}$ and $\psi_{j}$ states and the second-order matrix element $T_{021}^{2}=P_{02}^{2}P_{12}^{2}$.

On the other hand, another important concept is the Raman gain, which characterizes the efficiency of the Raman scattering process for producing coherent emerging radiation. For the gain formula to be obtained, it is necessary to calculate the nonlinear susceptibility $\chi^{\left(3\right)}$ given by the following [45]:

(12) $$\chi^{\left(3\right)}\left(E_{S},E_{L}\right)=-i\frac{\sigma_{01}{\left|M_{02}\right|}^{2}{\left|M_{12}\right|}^{2}}{\varepsilon_{0}\left[{\left(E_{2}-E_{0}-E_{L}\right)}^{2}+\Gamma^{2}\right]\Gamma }\;.$$

From this, the Raman gain can be obtained by using

(13) $$G_{R}\left(E_{S},E_{L}\right)=-\frac{\omega_{S}}{n\;c}Im\left(\chi^{\left(3\right)}\left(E_{S},E_{L}\right)\right){\left|E\right|}^{2}\;,$$

where $n=\sqrt{\varepsilon_{w}}$ is the refractive index, $\omega_{S}$ is the frequency of the secondary radiation, $\Gamma =ћ/\tau_{\mathrm{in}}$ is the line width of the susceptibility [39], and E is the amplitude of the pumping or incident field, which can be related to its intensity $I_{L}=1\times 10^{10}$ W m^−2^ [39] by ${\left|E\right|}^{2}=\frac{2I_{L}}{nc\varepsilon_{0}}$. The energy of the incident field $E_{L}$ can take a wide spectrum of values. It can be given by the physical process of electron excitation, in which the electron is supposed to be pumped into a virtual state $\psi_{2}^{\prime }$ with energy $E_{2}^{\prime }$ from the ground state, before lasing the secondary radiation [48]. This $E_{2}^{\prime }$ is related to the energy of the real state $E_{2}$ through the detuning $\delta =E_{2}-E_{2}^{\prime }=E_{2}-E_{0}-E_{L}=5\;\mathrm{meV}$ by which a value of $E_{L}=\max\left\{E_{2}-E_{0}\right\}-\delta$ is obtained.

Note that the parameter $n_{d}$ represents the nominal sheet doping density, while the actual carrier population in each subband is determined by the Fermi–Dirac distribution at finite temperature. Therefore, temperature effects are inherently included through the occupation factors, which control the population differences that enter the Raman susceptibility and Raman gain.

Variations in doping primarily modify the Fermi level position and consequently the occupation probabilities, but they do not alter the electronic eigenenergies of the QW. Since the Raman peak positions and spectral shifts are determined by intersubband energy differences, they remain unaffected by reasonable variations of carrier density. Only the amplitude of the absorption and gain spectra changes proportionally to the population difference.

## 3. Results

Within the use of licensed COMSOL Multiphysics software, the FEM was employed to obtain the wavefunctions and their respective energies in Equation (4) [49,50,51]. In the software, a user-controlled mesh was used for the calculation over the entire system with a maximum element size of 0.05 nm, a maximum element growth rate of 1.1, and a total of 441 evaluation nodes, without any mesh refinement. The other parameters were also obtained in COMSOL through parametric sweeps and the built-in integration function of the software.

A mesh convergence analysis was carried out to ensure numerical accuracy of the eigenenergies and dipole matrix elements. The maximum element size was progressively reduced and the calculated subband energies and transition matrix elements were compared between successive refinements. Convergence was reached when relative variations were below $10^{-4}$ for eigenenergies and $10^{-3}$ for dipole matrix elements. The adopted mesh (maximum element size of 0.05 nm with 441 evaluation nodes) satisfies these criteria, since further refinement produced negligible changes in the optical coefficients.

**Figure 3:**
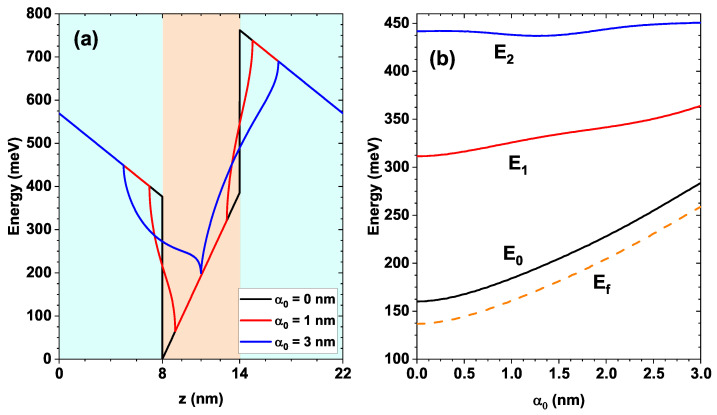
In (**a**), variation of the dressed confinement potential *V* along position on the *z*-axis in a ZnO/${\mathrm{Mg}}_{0.2}{\mathrm{Zn}}_{0.8}$O QW system with dimensions $L_{w}=6\;\mathrm{nm}$ for the well and $L_{b}=8\;\mathrm{nm}$ for the barriers. Different values of the dressing parameter α_0_ = 0 nm, 1 nm, and 3 nm are considered. In (**b**), variation of the energies for ground state $E_{0}$, first $E_{1}$ and second $E_{2}$ excited states, and Fermi level $E_{f}$ (dashed line), depending on the dressing parameter $\alpha_{0}$ in a ZnO/${\mathrm{Mg}}_{0.2}{\mathrm{Zn}}_{0.8}$0 QW system with dimensions $L_{w}=6\;\mathrm{nm}$ and $L_{b}=8\;\mathrm{nm}$.

The parameters used in the numerical calculations are listed in Table 1. Figure 3 shows the bottom profile of the conduction band for a QW ZnO with length $L_{w}=$ 6 nm, with equal Mg_0.2_Zn_0.8_O barriers of $L_{w}=$ 8 nm; additionally, the effects of internal electric fields $F_{w}$ and $F_{b}$ are included in both barrier and well regions. In this system, the effect of an intense non-resonant laser field determined by the dressing parameter $\alpha_{0}$ has been included. Figure 3a compares the modification of the potential profile of the system initially without a laser field (black curve), with a laser parameter $\alpha_{0}=$ 1 nm (red curve) and for $\alpha_{0}=$ 3 nm (blue curve).

Table 2 includes a quantitative result related to the variation of the quantum well bottom connected to the left barrier for different values of the laser parameter. As this parameter increases, a clear reduction in well depth is observed, significantly altering the confined states.

The potential modified by the effect of the intense laser field has been calculated according to Equation (5) and modifications are evident in the interfaces between both materials with a curved characteristic, which is a typical effect of the response to an intense non-resonant laser field; also, note the decrease in the depth of the central well that is proportional to the magnitude of the dressing parameter (compare the well region for $\alpha_{0}=$ 1 nm and $\alpha_{0}=$ 3 nm). The mentioned modifications result in specific changes in the system’s limited states, which imply potential control over its optical properties, as will be discussed later. This laser-induced symmetry directly affects the dipole matrix elements and, consequently, the allowed optical transitions.

Figure 3b shows the energy variation as a function of the laser parameter $\alpha_{0}$ for the first three confined states in the quantum well system with $L_{w}=$ 6 nm for the well and $L_{b}=$ 8 nm for the barriers. The ground state is represented by the black line, the first and second excited states are depicted by the red and blue lines, respectively, and the Fermi level is shown in dashed lines.

As the laser parameter increases, the bottom of the quantum well shifts toward higher energies, as shown in Figure 3a. This results in a blue shift of the energy levels, and the most affected state is the ground state since it is located closer to the bottom of the well. In the range of variation of the laser parameter $0.0<\alpha_{0}<3.0$, the ground state varies approximately within $174\;\mathrm{meV}<E_{0}<286\;\mathrm{meV}$.

Note that as $\alpha_{0}$ increases, the energy separation between the ground state and the first excited state decreases (see the black and red curves in Figure 3b). The same behavior occurs for the separation between the first and second excited states (see the red and blue curves in Figure 3b). This is because the ground state tends to increase its energy along with the bottom of the well. However, this effect is compensated by the fact that the well also increases its average width, causing the higher-energy states to shift downward and become closer in energy.

The laser-induced modulation mechanism is governed by the ratio between the laser parameter and the confinement length. Therefore, changing the well width or Mg composition mainly rescales the intersubband energies but does not modify the physical origin of the Raman gain modulation. A potential increase in the QW width would shift the electronic states to lower energies and reduce their transition energies. Similarly, by varying the Mg composition, the number of confined states in the QW useful for Raman analysis can be tuned.

Note that the characteristic electronic transition energy of the system is given by the intersubband separation $E_{10}$ (which varies between 75 meV and 150 meV in the range taken from the laser parameter), while the intense laser field is characterized by the photon energy $E_{L}$ (which will be taken as 276.61 meV and 400 meV). The present treatment assumes the high-frequency off-resonant regime, $E_{L}>>E_{10}$, ensuring that the laser field induces dynamical renormalization of the confinement potential rather than real electronic transitions.

**Figure 4:**
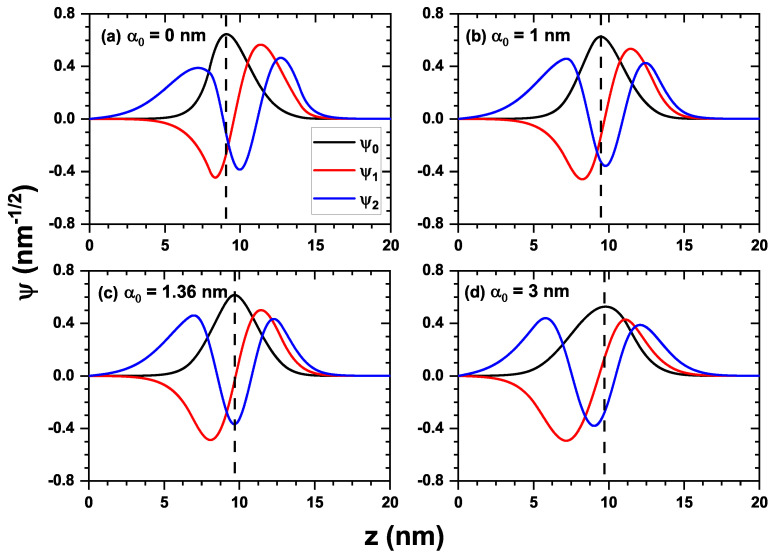
Normalized wave functions for the first three states for a ZnO/Mg_0.2_Zn_0.8_O QW system with dimensions $L_{w}=6$ nm and $L_{b}=8$ nm, depending on different values of the dressing parameter $\alpha_{0}$. The dashed line represents the point of maximum amplitude for the wave function $\psi_{0}$ in each of the values for $\alpha_{0}$. In (**a**), the dressing parameter value taken is $\alpha_{0}=0$ nm, while in (**b**) the parameter is taken as $\alpha_{0}=1$ nm. The value in (**c**) is obtained through the cancelation of the matrix element $M_{02}$, which gives $\alpha_{0}=1.36$ nm. In (**d**), the last value considered for the parameter is $\alpha_{0}=3$ nm.

Figure 4 shows the wave functions of the first three confined states in the quantum well: the ground state $\psi_{0}$ (black curve), and the first and second excited states $\psi_{1}$ and $\psi_{2}$ (red and blue curves, respectively), for four different values of the laser parameter: $\alpha_{0}=0$ in panel (a), $\alpha_{0}=1$ nm in panel (b), $\alpha_{0}=1.36$ nm in panel (c), and $\alpha_{0}=3$ nm in panel (d). The dashed line in each panel corresponds to the maximum value of the wave function associated with the ground state. This line has been included to analyze the symmetry of the states as the laser parameter increases. In Figure 4a, the system does not include the laser field ($\alpha_{0}=0$), and the wave functions are determined solely by the structure’s confinement potential. Note that the ground state exhibits a shift toward the left, caused by the asymmetry of the triangular-shaped well, as shown in Figure 3a (black curve). Evidently, this lack of parity is also reflected in the first and second excited states. Clearly, the ground state is not an even function, as indicated by the absence of symmetry with respect to the vertical dashed line located at its maximum value. This feature affects the associated optical properties, as discussed later. Figure 4b shows the same states for a laser field of $\alpha_{0}=1$ nm, whose associated potential is depicted in Figure 3a (red curve). Note that the ground state and the first excited state tend to behave as even states (see the black and red curves). This behavior is not observed for the second excited state (blue curve). In Figure 4c, the laser parameter for which the wave functions reach their point of maximum symmetry is shown (for $\alpha_{0}=1.36$ nm). This result can be corroborated by calculating the dipole matrix elements between the states, as illustrated in a later figure. This behavior can be explained by the fact that the laser field modifies the bottom of the potential, transforming it from a sawtooth-like profile (clearly asymmetric) into a V-shaped potential (symmetric), thereby inducing a local parity in the lowest states of the system. In Figure 4d, for a laser parameter of $\alpha_{0}=3$ nm, the previously mentioned local symmetry is broken again (as seen in Figure 3a, blue curve), causing the lowest states to lose their parity once more.

**Figure 5:**
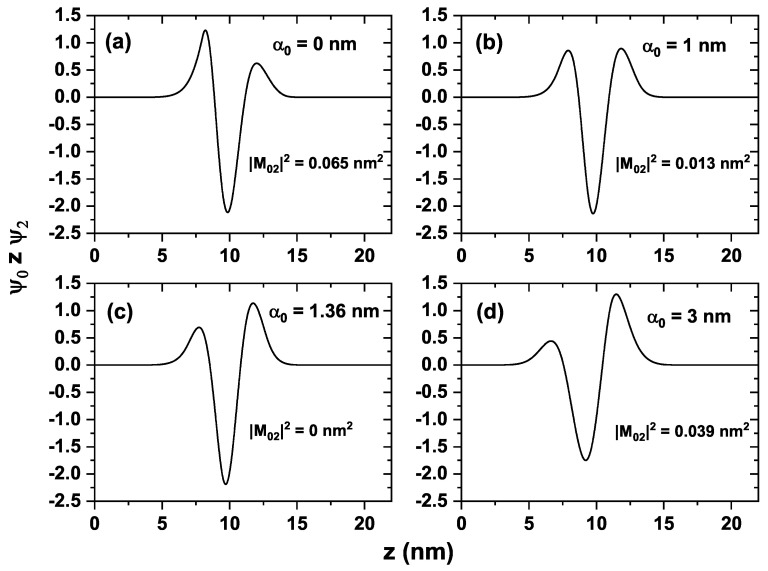
Argument of the matrix element integral $\psi_{0}z\psi_{2}$ plotted versus position *z* in the system for a ZnO/Mg_0.2_Zn_0.8_O QW system with dimensions $L_{w}=6$ nm and $L_{b}=8$ nm, considering different values for the dressing parameter $\alpha_{0}$. In (**a**), the argument for $\alpha_{0}=0$ nm is depicted, in which the left peak is higher than the right peak. In (**b**), the peak’s heights are equal for both left and right. In (**c**,**d**), this characteristic is inverted, since the right peak increases in height while the left peak decreases.

Figure 5 shows the integrand of the dipole matrix element $\psi_{0}\;z\;\psi_{2}$ as a function of the position *z* for four different values of the non-resonant intense laser parameter: $\alpha_{0}=0$, $\alpha_{0}=1$ nm, $\alpha_{0}=1.36$ nm, and $\alpha_{0}=3$ nm, corresponding to Figure 5a, Figure 5b, Figure 5c, and Figure 5d, respectively. Each figure presents the corresponding value of the squared dipole integral $|M_{02}{|}^{2}$, the highest value occurring for $\alpha_{0}=0$, where a clear asymmetry is observed in the local maxima of the curve (see Figure 5a). Subsequently, a significant decrease in the value is observed for $\alpha_{0}=1$ nm, where the local maxima reach similar values (Figure 5b). Figure 5c shows that the matrix element is zero for $\alpha_{0}=1.36$ nm, canceling the integral value, despite the fact that the two local maxima of the curve clearly differ. This is a characteristic of potential asymmetry, in which the area under the curve between the two maxima equals the area of the relative minimum. The cancelation of the integral arises from the compensation between the positive and negative contributions of the wave functions due to their spatial symmetry. Finally, for $\alpha_{0}=3$ nm, the matrix element again takes nonzero values, and the asymmetry of the system becomes more evident.

**Figure 6:**
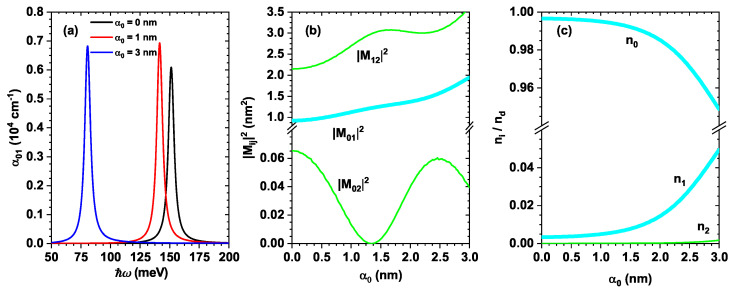
In (**a**), absorption coefficient $\alpha_{01}$ in the given ZnO/${\mathrm{Mg}}_{0.2}{\mathrm{Zn}}_{0.8}$O QW system with dimensions $L_{w}=6$ nm and $L_{b}=8$ nm, depending upon the incident photon’s energy ћω of the resonant laser for different values of $\alpha_{0}$. In (**b**), matrix elements $|M_{ij}{|}^{2}$, depending on the variation of the dressing parameter $\alpha_{0}$ for $\psi_{0},\psi_{1}\;\mathrm{and}\;\psi_{2}$ states in the given ZnO/${\mathrm{Mg}}_{0.2}{\mathrm{Zn}}_{0.8}$O QW system with dimensions $L_{w}=6$ nm and $L_{b}=8$ nm. In (**c**), normalized carrier density per unit area $n_{i}/n_{d}$ for the $\psi_{0},\psi_{1},\;\mathrm{and}\;\psi_{2}$ states in the given ZnO/${\mathrm{Mg}}_{0.2}{\mathrm{Zn}}_{0.8}$O QW system with dimensions $L_{w}=6$ nm and $L_{b}=8$ nm.

Figure 6a shows the linear optical absorption coefficient associated with the transition between the ground state and the first excited state, $\alpha_{01}$, for three different values of the laser parameter: $\alpha_{0}=0$, $\alpha_{0}=1$ nm, and $\alpha_{0}=3$ nm (black, red, and blue curves, respectively). Note that the peak corresponding to $\alpha_{0}=0$ is around 150 meV, which is consistent with the transition energy $ћ\omega =E_{1}-E_{0}\approx 150$ meV (see Figure 3, black and red curves). The magnitude of the absorption coefficient is proportional to the matrix element $|M_{01}{|}^{2}$, as shown in Equation (6), and its behavior as a function of the laser parameter is presented in Figure 6b. The red curve corresponds to $\alpha_{0}=1$ nm and exhibits a maximum at approximately 143 meV, which is in agreement with the energy difference between the states $E_{0}$ and $E_{1}$ for this value of the laser parameter (see Figure 3b). Note that this peak has a larger magnitude than the one corresponding to $\alpha_{0}=0$. This is due to the fact that the associated matrix element $|M_{01}{|}^{2}$ increases with the laser parameter, as shown in Figure 6b in the highlighted $|M_{01}{|}^{2}$ curve. Finally, the linear absorption curve for $\alpha_{0}=3$ nm (blue curve) reaches its maximum around 80 meV, which corresponds to the point where the states $E_{0}$ and $E_{1}$ are closest, as observed in the energy curve. The magnitude of this curve is slightly smaller than that for $\alpha_{0}=1$ nm, despite the fact that the matrix element reaches a higher value (on the order of 2 nm^2^), according to Figure 6b. This reduction is caused by the electronic population difference between the corresponding states, $\sigma_{01}=n_{0}-n_{1}$, which is also proportional to the linear absorption coefficient. Figure 6c shows this behavior, where it is evident that for $\alpha_{0}=0$ and $\alpha_{0}=1$ nm, the population difference remains very similar (with a clearly high occupation of the ground state); thus, in these cases, the magnitude of the absorption is mainly determined by the matrix element $|M_{01}{|}^{2}$. However, for $\alpha_{0}=3$ nm, the population difference reaches a minimum value, which explains the decrease in the corresponding absorption coefficient (for this value of the laser parameter, the electronic occupation of the ground state decreases slightly, and a relevant occupation of the first excited state begins to emerge).

According to Figure 6a, when the laser parameter increases from $\alpha_{0}=0$ to 3 nm, the absorption peak undergoes a red-shift from ћω≈150 meV to ћω≈81 meV. This behavior arises from laser-induced symmetry-breaking of the confinement potential. Within the Kramers–Henneberger picture, the intense non-resonant field replaces the static confinement U(z) by the laser-dressed potential $V(z,\alpha_{0})$, which corresponds to a spatial averaging of the well over the excursion amplitude $\alpha_{0}$. As $\alpha_{0}$ increases, the effective potential becomes wider and curved, as illustrated in Figure 3a. Consequently, the carrier wavefunctions become more delocalized, and the subband separation decreases, producing the observed reduction in the transition energy $E_{01}=E_{1}-E_{0}$ (Figure 3b). Simultaneously, the symmetry-breaking mixes the parity of the eigenstates, altering the overlap integral $M_{01}$, thereby increasing the oscillator strength and, consequently, the absorption amplitude. Hence, the spectral shift and the modification of the peak in Figure 6a are direct manifestations of the laser-induced reshaping of the quantum confinement rather than only a parametric variation of the dipole matrix element.

We have to note that excitonic effects in ZnO are indeed very important for interband optical transitions due to the large exciton binding energy. However, the present work addresses electronic Raman scattering associated with intersubband transitions within the conduction band of an n-doped QW.

Since both the initial and final states belong to the same band, no hole participates in the process and therefore bound electron–hole excitons are not formed. Furthermore, the presence of a two-dimensional electron gas screens the Coulomb interaction, strongly suppressing excitonic correlations at the considered carrier densities. Consequently, the Raman response is governed by single-particle intersubband transitions rather than excitonic resonances. For this reason, excitonic effects are not included in the model, which is consistent with standard treatments of intersubband Raman processes in doped semiconductor QWs.

**Figure 7:**
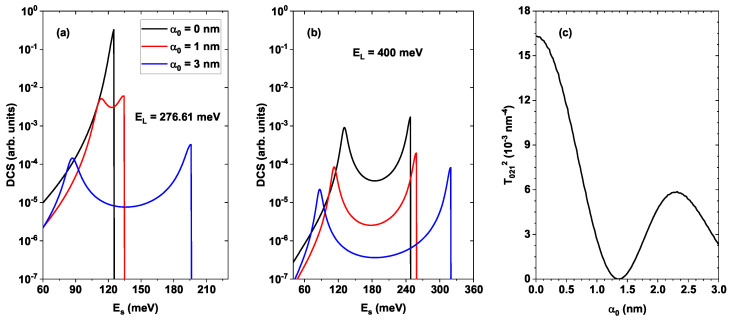
Raman differential cross section (DCS) in a ZnO/Mg_0.2_Zn_0.8_O QW with dimensions $L_{w}=6$ nm and $L_{b}=8$ nm, depending on the emitted radiation energy $E_{S}$ and different values of the dressing parameter $\alpha_{0}$. In (**a**), the value for the incident laser energy is taken as $E_{L}=276.61$ meV, given by the detuning δ=5 meV for the Raman transition process to the virtual state. In (**b**), the incident laser energy is set to $E_{L}=400$ meV for comparison. The picture in (**c**) shows the second-order matrix element $T_{021}$ depicted as a function of the dressing parameter $\alpha_{0}$.

Figure 7 shows the DCS (see Equation (9)) for the same previously fixed geometry and two different values of the laser pumping field, $E_{L}=276.61$ meV (Figure 7a) and $E_{L}=400$ meV (Figure 7b). The first value corresponds to the resonance energy between the second excited state and the ground state ($E_{2}-E_{0}-\delta \approx 276.61$ meV for $\alpha_{0}=0$). The second value of $E_{L}$ is much larger than this resonance in order to ensure proper excitation of the states involved. To better understand the behavior in Figure 7a–c, it has been included, showing the second-order matrix element $T_{021}$, which is proportional to the DCS.

The DCS must generate two peaks (resonant like and step like) because it is proportional to the transition rate, which is the product of two Lorentzian functions whose maxima are located at the positions $E_{s}=E_{2}-E_{1}$ and $E_{s}=E_{L}-\left(E_{1}-E_{0}\right)$ according to Equation (10). When $E_{L}=276.61$ meV (resonance point) and $\alpha_{0}=0$, the two maxima of the Lorentzian profiles are very close, differing only by a factor δ=5 meV. With the value of Γ used, the DCS behaves as a single peak with a value of approximately 125 meV (see the blue and red curves in Figure 3b), as shown in Figure 7a, black curve. This curve also exhibits a greater magnitude compared to the others, as the matrix element $T_{021}$ reaches a maximum value for $\alpha_{0}=0$, as can be seen in Figure 7c. For $\alpha_{0}=1$ nm (red curve in Figure 7a), two DCS peaks can already be resolved because the first resonance is located at the new position $E_{s}=E_{2}-E_{1}\approx 102$ meV and the second at $E_{s}=E_{L}-\left(E_{1}-E_{0}\right)\approx 276.61-\delta -147.5=124.1$ meV, with a smaller magnitude, as evidenced in the curve $T_{021}$ in Figure 6c for $\alpha_{0}=1$ nm. These transition energy values can be verified in Figure 3b. A similar situation occurs for $\alpha_{0}=3$ nm, where the DCS peaks are much farther apart because the resonances are now at $E_{s}=E_{2}-E_{1}\approx 87.5$ meV and $E_{s}=E_{L}-\left(E_{1}-E_{0}\right)\approx 276.61-\delta -81.3=190.3$ meV, respectively, and with an even lower magnitude for this value of the laser parameter, as can be seen from the $T_{021}$ curve.

In Figure 7b, the DCS and the peaks are much more clearly defined because the energy $E_{L}$ is far from the resonance energy between the states involved (significant detuning), allowing better resolution of the Raman peaks. However, the magnitude of these peaks decreases strongly because of the increase in the denominator of the Lorentzian functions in the transition rate. These results demonstrate the possibility of tuning the position and magnitude of the Raman peaks in ZnO/MgZnO quantum wells by modifying the characteristics of the non-resonant external laser field, which is essential for practical applications.

**Figure 8:**
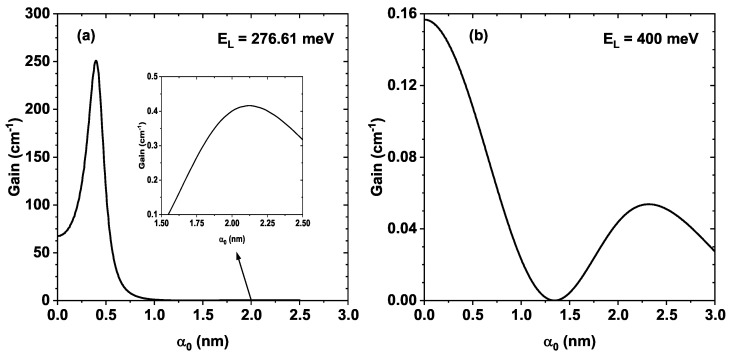
Raman gain for an electron in the given ZnO/${\mathrm{Mg}}_{0.2}{\mathrm{Zn}}_{0.8}$O QW system whose dimensions are $L_{w}=6$ nm and $L_{b}=8$ nm as a function of the parameter $\alpha_{0}$. In (**a**), the energy for the pumping laser is $E_{L}=276.61$ meV, which is obtained considering the detuning δ for the Raman transition; here, the gain curve is shown in a thinner range for $\alpha_{0}$ in which it is seen to have a dominant and well-defined peak near $\alpha_{0}=0.4$ nm. In (**b**), the energy for the pumping laser is taken as $E_{L}=400$ meV, and the curve, shown for the whole range for $\alpha_{0}$, is seen to have much less magnitude than in (**a**) and a very different form.

Figure 8 shows the Raman gain calculated from Equation (13) as a function of the laser parameter for the two values of $E_{L}$: 276.61 meV, which corresponds to the resonant case in Figure 8a, and 400 meV, which corresponds to the off-resonant case in Figure 8b. In the first case, Figure 8a, we have $E_{L}={\left(E_{2}-E_{0}\right)}_{\alpha_{0}=0}-\delta$; with this expression in the denominator of susceptibility, it takes the form ${\left(\left(E_{2}-E_{0}\right)-{\left(E_{2}-E_{0}\right)}_{\alpha_{0}=0}+\delta \right)}^{2}+\Gamma^{2}$, for $\alpha_{0}=0$, it becomes $\delta^{2}+\Gamma^{2}$. As the laser parameter increases, the term $E_{2}-E_{0}$ becomes smaller, reducing the denominator of susceptibility and increasing the gain, until it reaches a maximum value obtained when $E_{2}-E_{0}=\left(E_{2}-E_{0}\right)\alpha_{0}=0-\delta$ for approximately $\alpha_{0}=0.34$ nm. From this point onward, the susceptibility denominator increases, reducing the gain, as seen in Figure 8a. The gain is also proportional to the product of the matrix elements $|M_{02}{|}^{2}|{M_{12}}^{2}|$, whose behavior is shown in Figure 6b. This product becomes zero at approximately $\alpha_{0}=1.4$ nm and reaches a new maximum for $\alpha_{0}$ between 2 nm and 2.5 nm, as evidenced in the inset of Figure 8a.

Figure 8b shows the Raman gain for $E_{L}=400$ meV, that is, an energy much higher than the system’s resonance energy. This increase causes the term ${\left(E_{2}-E_{0}-E_{L}\right)}^{2}$ in the denominator of the gain to take very large values, producing a decrease throughout the entire range of the laser parameter. This explains the significant difference in magnitude between Figure 8a,b. In this figure (b), two local maxima are observed for $\alpha_{0}=0$ and approximately at $\alpha_{0}=2.4$ nm, which coincide with the local maxima of the matrix element $|M_{02}{|}^{2}$ in Figure 6b. Note that the position of the gain minimum located near approximately 1.4 nm is also predicted.

In the resonant case (Figure 8a), the laser parameter can significantly modify the Raman gain in this system, increasing it from approximately 65 cm^−1^ to nearly 250 cm^−1^ as the parameter varies from 0 to 0.4 nm, that is, almost four times the initial magnitude. However, the magnitude of the Raman gain can also be controlled through the external pumping laser. This implies that the Raman gain in this system is not fixed; it can be tuned by external fields, which is useful for potential applications in field-dependent optoelectronic devices, quantum gain modulators, or controlled Raman amplifiers.

Normally, depending on the values of the Raman gain, potential applications could be considered, such as modulators (0.1–1 cm^−1^) or amplifiers (1–10 cm^−1^). According to Figure 8, depending on the chosen $E_{L}$, it is possible to modulate the gain through the laser parameter, entering the modulator region for $1.5\;\mathrm{nm}<\alpha_{0}<2.5\;\mathrm{nm}$ or the amplifier region for $\alpha_{0}$ close to 1.0 nm. Therefore, the results for the studied system indicate potential practical applicability as modulators or amplifiers, as the gain values fall within the accepted range.

The calculated laser-induced shifts of the Raman resonance provide a direct estimate of the achievable spectral tuning range, while the linewidth determines the optical bandwidth. In addition, the characteristic response time is governed by the electronic coherence time (τ=ћ/Γ), which for Γ=5 meV corresponds to sub-picosecond operation speeds. Therefore, the results imply that the proposed mechanism could enable wavelength tuning in the meV range with ultrafast response times, compatible with high-speed photonic modulation.

It should be noted that realistic loss mechanisms, including electron—phonon scattering and thermal dephasing, are effectively accounted for through the broadening parameter Γ. An increase in these losses reduces the Raman gain magnitude, while the spectral position and the laser-field-induced tunability remain unchanged. Therefore, the predicted gain enhancement represents an upper bound that can be optimized experimentally by minimizing scattering and operating at low temperatures.

Although the calculations presented herein are restricted to a single ZnO/Mg_*x*_Zn_1−*x*_O QW geometry, the discussion of the physical mechanisms allows a broader interpretation. For example, variations in the well width mainly affect the energy difference between confined subbands, with wider wells leading to reduced intersubband transition energies and narrower wells enhancing quantum confinement. Additionally, Mg composition directly affects the conduction band offset and the internal electric fields arising from spontaneous and piezoelectric polarization. One can expect that increasing the Mg composition will strengthen confinement and internal fields, leading to larger energy differences between subbands and a noticeable modification of the potential profile. Thus, a systematic exploration of geometry and Mg composition parameters would provide additional quantitative optimization guidelines for device implementations and could be addressed in future work.

In doped QWs, many-body interactions mainly introduce collective corrections to the single-particle intersubband transitions, such as the depolarization shift and exchange-correlation contributions. These effects renormalize the transition energies and oscillator strengths but do not alter the physical mechanism underlying the laser-induced tunability. In particular, the intersubband transitions would be shifted to a collective excitation energy (including the transition energy between electronic states, as well as the energy correction due to exchange-correlation effects and many-body interactions), while the intense laser field modifies the confinement potential and therefore shifts the dressed energies in the same manner. Consequently, many-body effects would quantitatively alter the resonance position and peak amplitude, while preserving the predicted spectral tuning and gain behavior.

Intersubband transitions have been directly observed in ZnO/MgZnO heterostructures through photocurrent and absorption spectroscopy, reporting transition energies in the range of 250–410 meV at room temperature and showing good agreement with effective-mass calculations [52,53]. Moreover, the transition energies strongly depend on the well width, confirming the sensitivity of the confined electronic structure to external perturbations. External electric fields have also been experimentally shown to modify the absorption spectrum and enable otherwise forbidden transitions, demonstrating that the optical response can be dynamically tuned [54].

## 4. Conclusions

In this work, we investigated the effects of a non-resonant intense laser field on the optical absorption and Raman scattering processes in ZnO/Mg_0.2_Zn_0.8_O quantum wells by analyzing the modifications induced in the confinement potential, electronic structure, transition matrix elements, and nonlinear optical response; these types of heterostructure give rise to internal electric fields that have also been modeled. Our results demonstrate that the dressing parameter $\alpha_{0}$, which characterizes the strength of the external laser field, introduces significant and highly tunable modifications to both the energy levels and the spatial distribution of the confined electronic states. These laser-induced alterations lead to pronounced shifts in intersubband transition energies, symmetry-driven changes in the dipole matrix elements, and a strong redistribution of carrier populations.

The interplay between these effects results in substantial tailoring of the linear optical absorption coefficient and the Raman response. In particular, the Raman differential cross section exhibits controllable peak positions and magnitudes, directly related to the behavior of the second-order matrix element $T_{021}$. Likewise, the Raman gain shows a remarkable sensitivity to the laser field: under resonant pumping conditions, the gain can be enhanced by nearly a factor of four for moderate values of $\alpha_{0}$, whereas in the non-resonant scenario, the overall magnitude is strongly suppressed but still retains a field-dependent structure associated with the matrix-element modulation.

These findings demonstrate that the optical response of ZnO/MgZnO QWs is not a fixed property of the heterostructure but can be efficiently tuned by external non-resonant laser fields. The ability to modulate absorption, Raman scattering, and Raman gain through an accessible control parameter suggests promising opportunities for the design of field-dependent optoelectronic devices, tunable Raman amplifiers, quantum gain modulators, and other photonic technologies based on wide-bandgap oxide semiconductors. Typically, depending on the Raman gain values, potential applications may include modulators (0.1–1 cm^−1^) and amplifiers (1–10 cm^−1^). According to Figure 8, depending on the $E_{L}$ considered, it is possible to tune the gain through the laser parameter, reaching the modulator regime for $1.5\;\mathrm{nm}<\alpha_{0}<2.5\;\mathrm{nm}$, or the amplifier regime for $\alpha_{0}$ values close to 1.0 nm.

## Figures and Tables

**Table 1:** Parameters used for the ZnO/Mg_*x*_Zn_1−*x*_O QW calculation.

Parameter (Units)	Mg_*x*_Zn_1−*x*_O
Vacuum electron mass $m_{0}$ (kg)	$9.11\times 10^{-31}$
Reduced Planck constant *ћ* (eV s)	$6.58\times 10^{-16}$
Boltzmann constant $k_{B}$ (eV/K)	$8.62\times 10^{-5}$
Vacuum permittivity $\varepsilon_{0}$ (F/m)	$8.85\times 10^{-12}$
System temperature *T* (K)	300
Well internal electric field $F_{w}$ (MV/cm)	−0.24
Barrier internal electric field $F_{b}$ (MV/cm)	0.64
Band discontinuity $\Delta E_{c}$ (meV)	376.5
Electron effective mass $m^{*}$ ($m_{0}$) [39]	0.23+0.05x
Dielectric constant ε ($\varepsilon_{0}$) [39]	8.1+1.5x
Total polarization $P_{\mathrm{tot}}$ (C/m^2^) [39]	0.032x
Intrasubband relaxation time $\tau_{\mathrm{in}}$ (ps) [39]	0.24
Incident field’s intensity $I_{L}$ (W m^−2^) [39]	$1\times 10^{10}$
Energy bandgap $E_{g}$ (eV) [42]	3.37+2.51x
Superficial carrier density $n_{d}$ (cm^−2^) [43]	$8.5\times 10^{11}$

**Table 2:** Height difference between the bottom of the potential profile and the left-barrier extreme of the well in a ZnO/Mg_0.2_Zn_0.8_O QW for different values of the dressing parameter $\alpha_{0}$.

$\alpha_{0}$ Parameter (nm)	QW Depth (meV)
0	376.3
1	336.1
3	249.7

## Data Availability

The original contributions presented in this study are included in the article. Further inquiries can be directed to the corresponding author.
